# The Origin of Social Evaluation, Social Eavesdropping, Reputation Formation, Image Scoring or What You Will

**DOI:** 10.3389/fpsyg.2016.01772

**Published:** 2016-11-14

**Authors:** Judit Abdai, Ádám Miklósi

**Affiliations:** ^1^Department of Ethology, Eötvös Loránd UniversityBudapest, Hungary; ^2^MTA-ELTE Comparative Ethology Research GroupBudapest, Hungary

**Keywords:** social evaluation, eavesdropping, image scoring, reputation, negativity bias, positivity bias, third-party interaction, comparative psychology

## Abstract

Social evaluation is a mental process that leverages the preference toward prosocial partners (positivity bias) against the avoidance of antisocial individuals (negativity bias) in a cooperative context. The phenomenon is well-known in humans, and recently comparative investigations looked at the possible evolutionary origins. So far social evaluation has been investigated mainly in non-human and human primates and dogs, however, there are few data on the presence of negativity/positivity bias in client-cleaner reef fish interactions as well. Unfortunately, the comparative approach to social evaluation is hindered by conceptual and procedural differences in experimental studies. By reviewing current knowledge on social evaluation in different species, we aim to point out that the capacity for social evaluation is not restricted to humans alone; however, its building blocks (negativity and positivity bias) may be more widespread separately. Due to its importance in survival, negativity bias likely to be widespread among animals; however, there has been less intensive selective pressure for the identification of prosocial companions, thus the latter ability may have emerged only in certain social species. We present a general framework and argue that negativity and positivity bias evolve independently and can be considered as social evaluation only if a unified behavior and cognitive system deals with both biases in concert.

## Introduction

Individuals of many species form temporary or permanent groups. Living closely to conspecifics has benefits such as more efficient defense against predators or hunting, however, the competition over resources may also increase among group members. It is assumed that the recognition of group mates as prosocial or antisocial is important in many gregarious species to predict the future behavior of others (Bonnie and Earley, 2007; Subiaul et al., 2008; Bräuer, 2014). Thus this skill can contribute to survival due to the avoidance of harmful individuals and can facilitate the choice of an appropriate partner to engage in successful cooperation. In recent years, growing body of studies have been conducted on different species investigating whether individuals show different behavior toward prosocial and antisocial partners. The phenomenon was studied mainly in human infants (*Homo sapiens*), non-human primate species (e.g., chimpanzees, *Pan troglodytes*) and dogs (*Canis familiaris*). Although researchers used similar behavioral framework (cooperative context) they approached the question from different perspectives and used different terminology to describe the phenomenon. This makes it difficult to examine the evolutionary origin of social evaluation which we define as the ability to distinguish between antisocial and prosocial others and display different behavior toward them.

In human infants researchers often discuss the ability to distinguish between antisocial and prosocial partners in relation to moral development under the term *social evaluation* (Hamlin et al., 2007). According to Sheskin and Santos (2012), the results of these experiments suggest that human infants show an early form of morality concerning the harm domain (Haidt and Joseph, 2008) relatively early in ontogeny. Hamlin (2013b) argued that moral sense might have evolved to sustain cooperation. Thus social evaluation provides the mental support to discriminate between potential partners in a cooperative context. In studies with non-human primates researchers used a similar viewpoint considering the problem of choosing the appropriate partner to cooperate with and more importantly, to avoid harmful individuals. However, these authors used different terms to describe the phenomenon, such as reputation formation (Herrmann et al., 2013), image scoring (Russell et al., 2008) and social evaluation (Anderson et al., 2013a). Researchers working on dogs referred to this skill mostly as social eavesdropping (Marshall-Pescini et al., 2011). In conclusion, authors from different research areas use related concepts to describe similar phenomena that have probably similar function in the different species.

Here, our aim is to present a general framework for this skill, which we refer to as social evaluation. Further, we review experimental work concerning social evaluation from the viewpoint of our definition and criteria, including our current knowledge about the emergence of this skill in different species and point out possible problems in research. This general framework should facilitate comparative studies in different species, and we hypothesize that the ability of social evaluation is not necessarily restricted to primates (and maybe dogs), but it is also present in other gregarious species.

The evaluation of individuals based on their behavior can be formed after engaging in a face-to-face (direct) or in a third-party (indirect) interaction. Acquiring information about others’ behavior by direct interaction might be the best predictor of their future behavior; however, in certain situations it can have a high cost. Thus learning about others and their behavioral tendencies by observing an interaction between two or more individuals can be advantageous (cf. social eavesdropping; Bonnie and Earley, 2007). However, this type of social information may be more difficult to handle cognitively and it is less accurate due to the possible lack of information about the social relationships and rules that are present in the observed group (Subiaul et al., 2008).

Throughout the present review, we use the term *prosocial* for cooperative and helpful behaviors, and the term *antisocial* for partners that hinder another to reach its goal or act selfishly in a cooperative situation.

## General Framework for Social Evaluation

### General Criteria for Social Evaluation

Social evaluation is defined as a mental process during which an individual (1) assigns different values (positive, negative) to particular behavioral patterns (e.g., helping, hindering) that are performed in a social interaction (e.g., problem solving), (2) associates these behaviors with specific individuals (partnership values) and (3) shows different behaviors (e.g., avoidance or preference) toward others based on the overall value which has been associated with them.

Social evaluation is composed of two building blocks. Negativity bias refers to an aversion of negative (social) stimuli that can manifest in the avoidance of the antisocial partner (Hamlin et al., 2010; Anderson et al., 2013a; see below). In contrast, positivity bias is the preference toward positive (social) stimuli that can manifest in the preference toward the prosocial partner (Hamlin et al., 2007; see below). It is important to note that in the present review we use the terms negativity and positivity bias only in a cooperative context and as the manifestations of social evaluation, not in a general sense (Vaish et al., 2008). However, we include not only cooperation, but cooperative behavior as well, thus not only interactions that provide benefit to both parties, but also take into account the actions of a single individual toward the other (for the argument about the distinction between the two terms, see Bergmüller et al., 2007).

**FIGURE 1:**
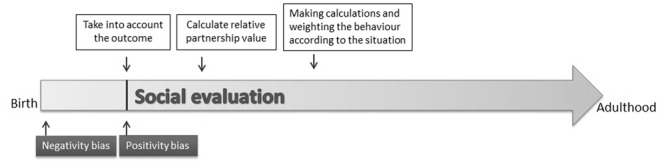
**Development of fully fledged social evaluation in humans.** According to Hamlin et al. (2010) negativity bias emerges soon after birth and at around 6 months of age positivity bias also manifests (Hamlin et al., 2007). Later infants are also able to take into account the context and the relative social roles of the partners (Hamlin et al., 2011). Based on results of Skowronski and Carlston (1987) at least adult humans weight the performed behavior of others when assigning partnership values to them.

In the simplest case, there can be three possible scenarios according to the behavior of the potential partners in which the subject may show its preference: positive vs. negative partners; positive vs. neutral partners; and negative vs. neutral partners. Based on the fitness consequences we expect that individuals choose the relatively positive partners, thus the prosocial partners in the first two, and the neutral partner in the third scenario. In the current literature researchers tend to refer to social evaluation even when individuals only show avoidance of a negative partner, but do not discriminate between a prosocial and a neutral agent (Anderson et al., 2013a). In contrast, we suggest that the term social evaluation should be restricted to cases when both negativity *and* positivity bias can be detected, that is, the individual shows clear preference in all three scenarios (**Table 1**). Our assumption is that negativity and positivity bias emerge independently from each other and evolve later to a unified behavior system, i.e., both abilities can occur without the presence of the other. Thus social evaluation can be considered as a hierarchical system, in which negativity and positivity biases are the building blocks that merge into a unified system. Social evaluation cumulates both negative and positive partnership values which results in the avoidance of or the preference toward others based on their overall partnership values. In some species social evaluation ability may also be able to take into consideration the context of the behavior and weight the importance of it to the overall value (**Figure 1**, see later).

**Table 1:** Manifestations of social evaluation and its building blocks in case of different partner-type arrangements.

Type of the partners			Response	
Partner		Partner	Building block	Phenomenon
A		B		
Prosocial	>	Antisocial	Negativity and/or positivity bias	–
Prosocial	>	Neutral	Positivity bias	Social evaluation
Neutral	>	Antisocial	Negativity bias	

We would like to emphasize that other features of the potential partner, for example, its skillfulness as a cooperator also have an effect on the individuals’ choice when recruiting a collaborator (Melis et al., 2006). We argue that both aspects (skillfulness and prosociality) are important in such contexts and the two cannot be separated sharply; however, they can be discussed independently (e.g., in case of two equally effective partners their level of prosociality can have an impact on individuals’ choice). In the present review, we will focus only on the importance of negativity and positivity bias as the basis of partner choice.

### The Two Facets of Social Evaluation

In the following, we summarize the two different types of manifestations of social evaluation, in terms of function, that is, how these skills may contribute to fitness. Based on the function of these biases we suggest that there is probably a stronger natural selection on negativity, than on positivity bias due to the higher cost involved in case of the former.

#### The Function of Negativity Bias

The aversion toward any type of negative stimuli that reduce survival is an advantageous skill; including those associated with social interactions. For example, capuchin monkeys (*Cebus apella*) are known to be prosocial and cooperate in many situations (de Waal et al., 1993; Mendres and de Waal, 2000). Thus one may expect that they have the ability to recognize and avoid antisocial others. In line with this Anderson et al. (2013a) found that in laboratory setting capuchin monkeys show aversion to an antisocial human partner compared to a neutral one after observing that the partner refused to help in a problem situation (see below).

Engaging in any kind of interaction with an antisocial agent can have harmful consequences, thus avoiding these individuals in general prevents the possibility of a bad outcome. Interacting with an unfamiliar individual about whom one does not have any information can also be risky. In these cases the situation itself has the main effect on the individual’s choice, i.e., if choosing an antisocial partner has higher cost than the advantage gained by choosing a prosocial individual then it is better to avoid to the unfamiliar partner. Thus the unfamiliarity of a partner can have a negative partnership value. For example, in bluestreak cleaner wrasses (*Labroides dimidiatus*) and their client reef fishes, it is more advantageous to the client to choose the known cleaner rather than an unknown one (Bshary and Grutter, 2006; see the details later).

#### The Function of Positivity Bias

In a general sense positivity bias is the preference toward positive (social) stimuli. As noted above, not being able to choose the (more) prosocial partner may result in smaller cost in terms of fitness. However, in long-term closed groups, preference for prosocial individuals may lead to significant gains. Thus one might hypothesize that in such species there would be a positive selection for this skill. Unfortunately, in most of the studies investigating the ability to discriminate between prosocial and antisocial partners we cannot distinguish between positivity and negativity bias due to the lack of comparison with a neutral partner. However, Hamlin et al. (2007) found that from at least 6 months of age human infants show preference toward the prosocial partner compared to a neutral one. In spite of the lack of evidence, we assume that in non-human species that live in closed, long-term social groups positivity bias might emerge as well.

## Comparative Approach to Social Evaluation

In the following, we review experimental studies which we have identified as tackling the same phenomenon, despite the fact that they may have been put forward on different theoretical basis and utilize a different terminology.

### Social Evaluation in Human Infants

In a series of studies, Hamlin et al. (2007, 2010, 2011) investigated whether human infants show different behavior toward prosocial and antisocial partners. Researchers used different inanimate agents (geometrical shapes or hand puppets) as social partners and presented an interaction between them to the infants. In these scenes a recipient needed help to reach a goal (e.g., get to the top of a hill, get open a box, etc.) and interacted with either a prosocial (helper), an antisocial (hinderer), or a neutral agent. In their first study, Hamlin et al. (2007) investigated whether infants of 6 and 10 months of age show some behavioral evidence for social evaluation. Infants observed an inanimate object trying to go up a hill and failing twice, and then another agent entered the scene. Based on their role the partner helped or hindered the recipient to reach its goal, and the neutral partner moved uphill or downhill without engaging in an interaction. Infants of both age groups chose the prosocial partner over the antisocial and the neutral agent and before reaching for the partner they looked longer at the prosocial than the antisocial agent (Hamlin et al., 2010). These results suggested that infants as young as 6 months are able to socially evaluate others in a cooperative context.

Some authors raised concerns about the method used by Hamlin et al. (2007) and argued that some aspects of the procedure might have influenced infants’ choice preference (Scarf et al., 2012; but see Hamlin, 2014b). However, Hamlin and Wynn (2011) obtained similar results in infants of different ages in other situations playing with hand puppets in front of the subject. In one of the experiments in the observed scene the recipient tried to open a box to obtain the toy from inside, but failed. Then either a prosocial partner helped to open the box or an antisocial agent shut it hindering the recipient to obtain the toy. In the other experiment, infants saw an agent playing with a ball and dropping it accidentally in the direction of one of two agents. After the agent grabbed the ball, the recipient asked for it. The prosocial agent gave it back to the recipient, but the antisocial partner took it offstage. In the first experiment they found that infants of 5 and 9 months of age choose the prosocial partner over the antisocial one and found the same choice preference in 5-month-old infants in the latter experiment as well. They also found that infants of 3 months of age look longer (‘preferential looking’) at the prosocial than at the antisocial agent in the social condition. However, in these cases a neutral partner was never introduced to the subjects, thus we do not know whether the observed discrimination between the agents was due to negativity and/or positivity bias.

In another study, Hamlin et al. (2011) used a similar procedure to test selective social evaluation in 5- and 8-month-old infants, i.e., whether the evaluation of others as prosocial or antisocial depends on the relative social roles played by the partners (relative partnership values). In this case subjects observed the toy-in-the-box scene described previously and then the ball-dropping play, but with the former prosocial or antisocial individual as the recipient. Authors found that while 5-month-old infants choose the prosocial partner in both cases regardless of the role of the recipient in the toy-in-the-box scene, 8-month-olds chose the antisocial partner, the partner that behaved antisocially toward an antisocial agent. Their results suggested that although younger infants only take into account the outcome of the interaction between third-parties, at least from 8 months of age they are able to evaluate the observed interactions globally.

Hamlin (2013a) also tested whether 5- and 8-month-old infants are able to evaluate others based solely on their behavior and not the outcome of their actions. In this study, they presented different scenarios to the subjects by presenting hand puppets in front of the infants as in previous studies. In the first experiment, the valence of the partners were different, but the outcome was the same from the viewpoint of the recipient (successful helper vs. failed hinderer; failed helper vs. successful hinderer). In the second experiment, the partners were a failed helper and a failed hinderer, thus the outcomes of their actions were contradictory with their individual social attitudes (e.g., failed hinderer is antisocial, but the outcome is positive). In the third case, they presented interactions in which the two partners had the same status, however, the outcomes were different (e.g., successful helper vs. failed helper). In all experiments infants could choose between the partners (two puppets) presented to them after the display. Only 8-month-old infants distinguished between the partners in the different experiments based on their behavior and not simply the positive or negative outcome of their actions, meaning that they chose the prosocial partner over the antisocial one. However, Hamlin (2014a) later found that when enough time is given to 5-month-old infants to process the interactions, they are also able to take into account the context in the evaluation of others.

The above results suggest that social evaluation emerges in at least 5–6 months old human infants, and one study showed that 3-month-olds also show preference toward the prosocial agent over the antisocial (Hamlin and Wynn, 2011). So far only one study investigated whether at 3 months of age positivity and/or negativity bias can be detected in infants. Hamlin et al. (2010) presented the ‘hill paradigm’ (see above; Hamlin et al., 2007) to 3-month-old infants and used preferential looking to measure whether they discriminate among prosocial, antisocial, and neutral agents. They found that infants this young looked longer at the prosocial than the antisocial partner, however, they did not differentiate between the prosocial and the neutral agent. In contrast, they looked four times longer at the neutral than the antisocial partner. Although a few months later infants showed preference toward a prosocial agent and apparently were able to take into account contextual information as well, at 3 months of age negativity, but not positivity bias can be detected. Based on this result authors concluded that the ability to socially evaluate others is a fundamental capacity; however, according to our criterion without the emergence of positivity bias this cannot be considered as social evaluation.

There was only one study in which researchers used a ‘sharing’ situation as the observed interaction in human infants (Herrmann et al., 2013). This is unfortunate from the comparative viewpoint because in non-human species researchers mainly used this particular situation to test social evaluation ability. Herrmann et al. (2013) first tested 30-month-old toddlers in direct interaction with two human partners and later presented a third-party interaction involving the same two humans now behaving toward a recipient. The human partner was either prosocial and tried to give a ball to the recipient (the toddler in the direct and an adult human in the indirect interaction), or antisocial who prevented the transfer by the prosocial human. Then both partners offered a ball to the toddler who could reach for one of them. The same moment the toddler reached for the ball, the partners withdrew their hands. The researchers found that after engaging in direct interaction with the partners toddlers show preference toward the prosocial human, while after watching the third-party interaction they did not show preference toward either of the partners. Note, however, in the latter case they had negative direct experience at choice (both humans withdrew their hands when the toddler reached for the ball) which probably had greater influence than evaluating them based on their behavior toward others. Thus, we cannot separate the effect of direct experiences with the partners from the results in the third-party interaction. It is also worth to note that although these results suggest that 30-month-old toddlers have difficulties in discriminating between prosocial and antisocial others in a third-party interaction, Hamlin et al. (2007, 2011) and Hamlin (2013a) found that infants as young as 5–6 months of age discriminate between prosocial and antisocial partners.

Hamlin et al. (2011) tested also 19- and 23-month-old toddlers in the box-opening and ball-playing scenario with hand puppets as partners. In this case in the test phase toddlers either got one treat to give it to one of the partners or had to take one treat from one of the partners. They found that toddlers of both ages gave the treat to the prosocial partner and took one from the antisocial one. Another research group found different results as Hamlin et al. (2011) when using adult humans as partners in a similar set up as the ball-playing scenario (Dahl et al., 2013). In this study, 17-, 22-, and 26-month-old toddlers observed an interaction between three humans. The experimenter (recipient) invited the partners to play and rolled them a ball. After the recipient asked them to roll it back, the prosocial partner did at request, but the antisocial human put it away. In the test phase the two partners both tried to reach the same object and the infants were allowed to help them. Dahl et al. (2013) found that infants of 17 and 22 months of age help equally often to the partners and only at the age of 26 months they prefer to help the prosocial partner over the antisocial human.

The results of the studies by Hamlin et al. (2010, 2011) and Hamlin (2013a) suggest a development of social evaluation in human infants: first only negativity bias can be detected and around 5–6 months of age positivity bias emerges, making the functioning of a social evaluation system complete. However, based on the results of Dahl et al. (2013) and Herrmann et al. (2013) we should be careful to draw conclusions about the functionality of this system at a very young age.

There are main differences between the studies described above. Hamlin et al. (2007, 2011) used inanimate objects as partners and presented different helping situations to infants of different ages, while the other two research groups tested older infants and used humans as partners. Furthermore, Herrmann et al. (2013) used a sharing situation instead of the helping scenario. Thus methodological differences across studies could be responsible for these divergent findings (see Discussion).

Recently, Salvadori et al. (2015) attempted to reproduce the study of Hamlin and Wynn (2011) by using the same procedure with slight modifications, but they failed to find the same results. Although such studies (Dahl et al., 2013; Salvadori et al., 2015) weaken the findings of Hamlin et al. (2007) and Hamlin and Wynn (2011), it should be take into consideration that the latter group found similar behavior in different contexts in children of different ages. However, there is a need for further studies to strengthen the latter findings.

### Comparative Experiments with Non-human Species

Other than humans the phenomenon has been studied only in non-human primate species, dogs and in cleaner-client reef fish interactions. In non-human animals, the ability to discriminate between prosocial and antisocial others was investigated from different perspectives and researchers used different, but related terms to describe the phenomenon. In this overview, the conceptual approach of the original experimental studies provided the basis of the categorization. In the following, we use the terms negativity/positivity bias and social evaluation in the above meaning, and refer to the terms used in the original studies only in the subtitles.

#### Reputation Formation in Non-human Apes

Researchers tested chimpanzees, bonobos (*Pan paniscus*), gorillas (*Gorilla gorilla*) and orangutans (*Pongo pygmaeus*) in different food sharing situations (Russell et al., 2008; Subiaul et al., 2008; Herrmann et al., 2013). In these studies, researchers used similar procedures in which subjects observed a human (or a conspecific) begging for food from two persons. In all cases, the prosocial partner gave food to the recipient (beggar), but the other partner either refused to share (Russell et al., 2008; Subiaul et al., 2008) or prevented the prosocial human to give food to the recipient (Herrmann et al., 2013). After the observation phase subjects were allowed to choose between the partners who offered food to them. Upon subject’s request either the prosocial partner gave and the antisocial partner refused to give food (Subiaul et al., 2008) or neither of the partners shared food (Russell et al., 2008; Herrmann et al., 2013).

There are two experiments (Russell et al., 2008; Subiaul et al., 2008; Experiment 1) that are mostly comparable with the studies conducted with human infants. In Russell et al. (2008) chimpanzees observed a third-party interaction between three humans. They found that only chimpanzees, but not bonobos, orangutans and gorillas prefer the prosocial human over the antisocial partner after observing a food sharing situation between them and a human recipient.

Unfortunately, in Russell et al. (2008) there is no information about the results of the critical first trial (whether there is a significant difference between times spent next to the two partners). However, subjects had direct experience with the partners during this trial which could influence their behavior toward the humans later. Considering that in chimpanzees there was a decline in time spent next to the antisocial partner over the trials, it would be interesting to see whether there was a significant difference between times spent next to the two partners in the first trial.

Furthermore, because the antisocial partner did not simply refuse to give, but also hit the recipient when he tried to reach the container, subjects might have been simply afraid of the antisocial human and this is why they avoided her. Considering that there is a decline over trials in approaching the antisocial partner, but there is no increase in the case of the prosocial partner, we can conclude that chimpanzees might show negativity, but not positivity bias in this context.

Subiaul et al. (2008) tested the same chimpanzee subjects in three experiments relying on the same procedures. In the first experiment, in which two unfamiliar humans behaved prosocially or antisocially toward a familiar human, none of the chimpanzees showed any preference toward the prosocial human partners after 16 trials. Thus, in the second experiment subjects received an extensive training to choose the prosocial partner after direct interaction. They were tested in blocks of eight trials and reached the criterion when they chose the familiar prosocial human at least seven times out of eight in two consecutive blocks. Four chimpanzees (out of seven) who reached the criteria showed significant preference toward both prosocial familiar and unfamiliar humans in subsequent tests. In the third experiment the same four subjects observed eight pairs of unfamiliar human partners interacting with another chimpanzee (six trials with each pairs of partners; third-party interaction). Subjects were tested in six trials with each pairs of partners and if they chose the prosocial one they obtained food from them (were reinforced for the correct choice), but not if they chose the antisocial partner. In the first test trials with the different partners the mean preference toward the prosocial unfamiliar human was 53%. However, in case of three out of four subjects during the first four sessions (24 trials with four different pairs of partners) the choice of the prosocial partner increased to 75%.

Thus it seems that chimpanzees in Subiaul et al. (2008) discriminated between prosocial and antisocial human partners only after receiving specific training. Furthermore, in the third experiment subjects not only could rely on their earlier direct experiences with partners showing similar behavior, but also during testing they received reinforcement to choose the prosocial partner (direct interaction). Thus it seems that chimpanzees do not show spontaneous discrimination between prosocial and antisocial human partners after indirect experience.

In their first experiment, Herrmann et al. (2013) exposed subjects (apes and 30-months-old human children) to a prosocial partner who attempted to offer them a piece of food, when the antisocial partner interfered and prevented the action by taking the food away (direct experience). After directly interacting with the partners orangutans and human children (see earlier) chose the prosocial partner more often, however, not chimpanzees and bonobos. In the next experiment the same subjects observed the same partners behaving the same way toward a third human (third-party interaction). In the test phase, both partners offered a piece of food to the subjects, but when they reached for it, both of the partners withdrew their hands. In this case orangutans and chimpanzees, but not bonobos and human children, preferred the prosocial partner.

However, some elements of the procedure used by Herrmann et al. (2013) could influence subjects’ choice. For example, as a response to the stealing by the antisocial partner the prosocial partner expressed her frustration by hitting and pushing the antisocial partner. This behavior of the prosocial partner could have elicited avoidance behavior on the part of the subject, thus the nature of this partner was not necessarily considered as prosocial.

In most of these experiments, subjects engaged in direct interaction with the partners before the third-party interaction, thus we cannot separate the effect of this experience from their results in the indirect context. Based on these it is still unclear whether chimpanzees and orangutans discriminate spontaneously between a prosocial and an antisocial partner after witnessing a third-party interaction.

Further problem is that only prosocial and antisocial partners were presented to the subjects. Thus even evidence for discrimination between the partners would not fulfill our definition of social evaluation.

#### Social Eavesdropping in Domestic Dogs

Recent findings suggest that dogs are able to coordinate their actions with each other and humans in cooperative tasks (Bräuer et al., 2013; Ostojić and Clayton, 2014). Thus showing avoidance of antisocial partners and/or preference toward prosocial ones can be advantageous in this species. Many dogs live in multi-human/dog groups, and get often in contact with other people. Thus considering that humans provide them different resources, it would be advantageous to be able to obtain information by observing their interactions with others. Domestic dogs were tested in food sharing situations to find out whether they show different behavior toward two human partners based on their behavior toward a third-party (Marshall-Pescini et al., 2011; Nitzschner et al., 2014; see below in detail). There was only one study in which dogs were tested in a helping situation (Chijiiwa et al., 2015; see below in detail).

Marshall-Pescini et al. (2011), Freidin et al. (2013), and Nitzschner et al. (2014) used a similar food sharing situation as described for non-human primates. Although the results of the experiment by Marshall-Pescini et al. (2011) showed that dogs prefer the prosocial human partner over the antisocial partner, the latter two studies suggested that dogs’ choice was influenced by the location of the human (in the test situation) rather than the identity and behavior. Kundey et al. (2011) tested dogs in a similar third-party interaction. However, they varied several elements of the procedure, for example, in one experiment either the recipient was an inanimate self-propelled object or the human partners sat inside boxes (they were invisible). The unfamiliar prosocial partner gave food to the recipient by placing it in front of her, while the antisocial partner put it front of the recipient and when she reached for it, the partner removed the food (prevented the recipient to take it). They found that dogs reliably choose the prosocial partner across experiments over the antisocial one, thus suggested that they are able to evaluate others based on their behavior in a social context. Considering that dogs showed this choice even when the “partners” were two boxes or the human partners behaved toward an inanimate object, dogs might simply associated the exchange of food with one of the partners and did not evaluate them based on their behavior toward another.

Nitzschner et al. (2012) conducted a study presenting a different situation than sharing food. Dogs observed an interaction between two human partners and a dog; the partner was either a nice human who behaved friendly with the demonstrator dog or a human who simply ignored the dog. Even though after a direct interaction subjects spend more time next to the nice experimenter, after the third-party interaction dogs did not show different behavior toward them. We suggest one alternative explanation other than raised in the original paper that can explain dogs’ behavior in this latter case. From the viewpoint of the demonstrator dog the humans could be considered as nice or ignoring, however, the subjects (witnessing the human-demonstrator interaction) were ignored by both humans (direct experience). We suggest that this direct experience could have greater effect on dogs than the information gathered in the third-party interaction.

Recently Carballo et al. (2015) investigated further whether dogs are able to discriminate between prosocial and antisocial humans after direct experience. The humans pointed at one of two bowls in front of the dog that contained food. However, while the prosocial partner let the dog eat the food, the antisocial partner took the food and ate it as soon as the dog found it. If there was a gender difference between the partners then dogs discriminated between them after six trials; 12 trials were needed for similar performance if both partners had the same gender (females). Thus it seems that even in the case of direct interaction dogs need more experience with human partners to discriminate between them based on their behavior.

Chijiiwa et al. (2015) implemented a helping scenario similar to the one used by Anderson et al. (2013a; see the next section). Dogs observed that their owner tried to open a container, but failed and requested help from one of the two human partners who (depending on the experimental group) either helped (prosocial) or refused to help by turning her head away (antisocial). The other (neutral) partner sat there quietly without engaging in any interaction. In a control group the owner stopped manipulating the object and then one of the human partners turned her head away. Thus only one of the partners was active during the demonstration and the owner interacted with this partner only (no interaction in the control condition). In the test trials dogs in the control group chose randomly. Dogs did not show any preference for the prosocial vs. neutral (non-active) partner. In contrast, dogs avoided the antisocial partner in favor of the neutral one.

However, several aspects of the procedure could have influenced the results of Chijiiwa et al. (2015). In case of Anderson et al. (2013a; see details later) the neutral partner was the recipient, thus subjects could see the action of the neutral partner as well, while in Chijiiwa et al. (2015) the neutral partner did not show any behavior during the observation phase (no interaction with the recipient). Thus the only available information about this partner was that she offered food during the test trials (prosocial act in direct interaction with the subject). Also, in the four test trials dogs had direct interaction with the partners who gave food to them upon request (including the neutral and antisocial partner). Thus information about the partners obtained during observations and in test trials were contradictory for some partners and may have influenced the results. Importantly, recalculations of the original data showed no significant difference between conditions in the first test trial^1^, i.e., no difference in subjects’ choice between the ‘active’ (prosocial, antisocial, and control) and the neutral partners. It is also worth to mention that in this study the recipient was the subject’s owner, thus the recipient/owner could carry over some contextual information from everyday life (interactions between the owner and others) to the situation used in the study.

In sum, although several studies were conducted with dogs it is not clear whether they adjust their behavior toward the partners based on their behavior toward others in a third-party interaction. Furthermore, due to the procedural arrangements we do not have clear information about the emergence of social evaluation or its components.

#### Social Evaluation in Capuchin and Marmoset Monkeys

In capuchin monkeys, Anderson et al. (2013a) used a problem solving situation to investigate social evaluation. Capuchins could witness two human partners who sat next to each other. One of them was always the recipient who tried to open a container to obtain an object from inside. In different trials (within subject design) the other person helped, refused to help, tried to help, but failed, or simply failed to acknowledge the help requested by the other person. After having observed the interaction between two partners, the capuchins could choose from whom they accept a piece of food. The recipient served as a neutral partner considering that she did not behave prosocially or antisocially. The capuchins chose the neutral partner over the antisocial partner in all cases, however, they did not show preference toward any of the humans when the partner helped, tried to help or was occupied during the request (was prosocial or neutral). These results suggest that capuchin monkeys show negativity, but not positivity bias that is not considered as social evaluation according to our criterion. This result is similar to that of 3-month-old infants who also showed negativity, but not positivity bias after observing a helping interaction between inanimate agents (Hamlin et al., 2010). However, we should note, that this refers to the mean performance reported after repeated experimental trials and we also have no information about capuchins’ choice in the first trial.

In a different study, Anderson et al. (2013b) investigated whether capuchins discriminate between two humans after observing a third-party reciprocity situation. Subjects observed a ball-transfer between two humans; *A* requested balls and *B* gave her own, and later *B* asked them back. In this case *A* either reciprocated or refused it by turning her head away. Then the partners offered food for the subjects who were allowed to choose from which of the partners they accept it. Capuchins did not show preference toward either of the partners after witnessing a reciprocated action by *A*, however, they chose the prosocial partner over the antisocial human after the non-reciprocated interaction. We suggest that in contrast to Anderson et al. (2013a), the recipient in this case was not a neutral agent considering that at the beginning of the interaction *B* transferred balls to *A*, thus it behaved prosocially. Based on this, capuchins observed an interaction between two prosocial partners, and between a prosocial and an antisocial partner. Thus the avoidance of the antisocial partner in the second scenario was not necessarily due to negativity bias and we cannot exclude the possibility of the presence of positivity bias either.

Kawai et al. (2014) investigated sensitivity to third-party reciprocity in common marmosets (*Callithrix jacchus*). Similarly as in Anderson et al. (2013b) in case of the reciprocal event the two humans (*A* and *B*) exchanged food, while in the non-reciprocal condition there was an initial transfer from *B* to *A*, but *A* refused to give food to *B*. Their results were similar as with capuchin monkeys (Anderson et al., 2013b), subjects were willing to accept food from both partner in the reciprocity condition; however, they preferred partner *B* in the condition where *A* refused to transfer. However, because of the similarities in the procedures the same problem arises here as in Anderson et al. (2013b), meaning that no neutral partner was introduced to the subjects and thus discrimination between the partners could be due to negativity and positivity bias as well.

Thus it seems that in capuchin monkeys at least negativity bias can be detected, however, we do not have convincing data on whether they show positivity bias as well, thus whether they are able to evaluate others socially. In case of marmoset monkeys we are also lack of information about the presence of negativity and/or positivity bias.

#### Reputation Formation in Cleaner-Client Reef Fish Interactions

The formerly described species are either share the same evolutionary clade with humans (non-human primates) or live in close contact with them (dogs). However, we suggest that similar capacity might be shown in non-mammalian species as well. Bshary (2002) and Bshary and Grutter (2006) conducted studies on bluestreak cleaner wrasses and their client reef fish species. Cleaners remove the ectoparasites from their client fish, however, some of them might consume the mucus and thus become a parasite instead of a cooperator. Clients also have the opportunity to eat the cleaner fish, however, this is less likely (Bshary, 2002). Thus for the client it is crucial to predict the prosocial or antisocial nature of the unfamiliar cleaner fish. In their study Bshary and Grutter (2006) used a procedurally similar method to that used in primates and dogs. They found that clients spend significantly more time next to a cleaner that they had the chance to observe while cooperating than next to a cleaner that did not engage in an interaction with the model client fish. In this case the second partner can be considered as a neutral partner and the cooperative cleaner as a prosocial individual, thus the choice behavior of the clients can refer to a positivity bias. However, in this case choosing an ‘unknown’ partner who might be antisocial has high cost. Thus clients might simply avoid the potential antisocial partner, thus the potential harmful consequences. The results of Tebbich et al. (2002) strengthen this assumption. They found that during the first 2 min of an experiment cleaner fish spends more time in the proximity of a familiar than an unfamiliar client (*Ctenochaetus striatus*). In the field, Bshary (2002) also found that clients were more likely to choose (invite for inspection) the cleaner whose previous interaction was positive than the one that was chased off by the client or its client darted off (negative interaction). However, if there was a longer time lag (>5 s) clients’ choice was not affected by the previous interaction of the cleaner. Unfortunately, in this situation the client could choose only between prosocial and antisocial partners, thus it is not possible to establish whether the skills of these fish fulfill the criteria of social evaluation. It would be interesting to examine the behavior of the clients when presenting them an interaction between a prosocial, antisocial, and ‘unknown’ partner in different arrangements to see whether we can detect negativity and/or positivity bias.

These results suggest that although currently the phenomenon is mainly studied in primates and dogs, it would be interesting and useful to conduct similar studies on other taxa as well.

## Discussion

### Comparison of Studies with Non-human and Human Animals

#### The Functional Concept of Social Evaluation

The investigation of social evaluation from an evolutionary and comparative perspective indicates that there is a need for general agreement on terminology and definition. We put forward that the concept of social evaluation should be reserved for assigning partnership values to other individuals in the group independent from its direction (negative or positive), although it is clear that avoiding others in such context is a more prevalent skill in animals. Accordingly, social evaluation allows the individual to enhance its fitness by being able to choose its partners for cooperative interaction based on direct or indirect (third-party) experience. This functional definition seems to provide a general framework for comparing different clades of animals that depending on the evolutionary history and ecological status may or may not display this skill.

Unfortunately, the possibilities of comparative investigations are limited but from an ecological and phylogenetic viewpoint non-human apes could provide an opportunity to establish specific hypotheses in relation to the manifestation of social evaluation. The few surviving species live in a variety of social organizations in which the significance of social evaluation could be different. Chimpanzees and bonobos live in large complex groups in which individuals engage in cooperative and competitive interactions, develop friendships (Silk, 2002, 2003) and form also coalitions (de Waal, 1984). In contrast the hierarchical groups in gorillas typically consist of a female harem and their offspring led by an adult dominant male (Robbins et al., 2004). Finally, orangutans are solitary for most of their life; many females live alone or with their offspring on non-overlapping territories of males (Singleton and van Schaik, 2002).

Thus based purely on their ecology we may predict that a well-developed ability of social evaluation should contribute to higher fitness in chimpanzees and bonobos, while its role in gorillas and orangutans could be negligible. Alternatively, fully functional social evaluation may have evolved much earlier in primate evolution and its manifestation depends on epigenetic factors (Chapman and Rothman, 2009). In this case testing captive individuals of these species may not provide meaningful insights because the social structure of these groups may differ from that in nature (Brent, 1992; Boesch, 2007), and their socialization to humans may also influence the development of social evaluation.

#### Contradictory Results, Methodological Issues

Researchers conducted similar studies with non-human primates and dogs both from the viewpoint of partners and situation (Russell et al., 2008; Marshall-Pescini et al., 2011); however, the results were contradictory in most of the cases. For example, in chimpanzees Russell et al. (2008) found that they show preference toward the prosocial human partner over the antisocial one after observing third-party interaction, however, the results of Subiaul et al. (2008) suggest that even in direct interaction it takes several trials for chimpanzees to choose the prosocial partner. In contrast, in Herrmann et al. (2013) chimpanzees did not discriminate between the partners in a direct interaction, only after observing their behavior toward another human later (indirect interaction).

It is also interesting that human infants’ performance seems to be influenced by the method used. In case of human infants and toddlers the set up that was used most often included inanimate objects as partners, and different helping situations were demonstrated to the subjects (Hamlin et al., 2007; Hamlin and Wynn, 2011). Despite some differences in details (e.g., context-dependency) the results show that social evaluation emerges relatively early in humans at around 5–6 months of age. However, when human adults were used as partners, even in a similar situation, researchers failed to find discrimination between the partners in 17- and 22-month-old toddlers (Dahl et al., 2013); they only found discrimination between the prosocial and antisocial human partners in 26-month-old toddlers. Herrmann et al. (2013) also failed to show discrimination between human partners in 30-month-old toddlers in third-party interaction using a sharing situation. There was only one study in which helping situation was used in non-human primates. Anderson et al. (2013a) found evidence for negativity bias in capuchin monkeys, and the findings of another study by Anderson et al. (2013b) also suggest that capuchin monkeys show negativity and/or positivity bias after observing the interaction between two human partners.

Thus it is likely that the different procedures measure different aspects of social evaluation in human children, and this should be clarified before these data are compared to those obtained from non-human animals. However, an important issue should be considered here as well: helping and sharing are two distinct domains (Warneken and Tomasello, 2009), and the presence of one does not indicate the functioning of the other. For example, it has been found that both human children and chimpanzees show helping behavior, but only the former tend to share resources with others (for a review, see Warneken and Tomasello, 2009). In light of this, we suggest that it is unfortunate that helping has been used most often when investigating social evaluation in children, but sharing situations in case of non-human species. Thus, we propose that in future studies this issue should be taken into account as well.

One might argue that in non-human primates and dogs using heterospecific partners could affect the outcome. However, companion dogs spend their life with humans and the tested non-human primates were also socialized to humans. Due to their extensive experience with humans it was not necessarily disadvantageous to use humans as partners in these studies but further comparative studies are warranted.

However, former experiences with conspecifics and/or humans and subjects expectation toward them can influence subjects’ behavior in studies investigating social behavior and cognition (Krause et al., 2011; Gergely et al., 2015). Several studies with domestic dogs support this assumption (Szetei et al., 2003; Erdõhegyi et al., 2007; and see also Topál et al., 2009) in which researchers showed that some aspect of dogs’ cognitive skills can be masked by using human social communicative cues and thus false conclusions can be drawn about their cognitive abilities. Utilization of unfamiliar moving inanimate objects (robots) as social partners can be useful in such investigations especially if the physical appearance of the robot does not resemble the embodiment of the subject species or any heterospecifics with whom they engage in daily interactions (Gergely et al., 2013). This way the test itself can be controlled by the experimenter more efficiently and it is also less likely that the robotic partner induces aversion. The novelty of the social partner hinders the possibility that subjects have any preliminary assumption about how the partner should behave in a given context (separation of expectations and the displayed behavior). The results of studies conducted with human infants using unfamiliar inanimate objects (Hamlin et al., 2011) and humans (Dahl et al., 2013) as partners also suggest that utilization of robots in such investigations can be advantageous.

Although, in the present review our main concern was about the emergence of social evaluation in third-party interactions, we would like to emphasize that subjects’ direct experience is also important (see below). Unfortunately, to our knowledge no studies have been conducted in which researchers would have systematically tested and compared the role of direct and indirect experiences in this context.

To investigate such questions, experimenters would need to control subjects’ experiences with other individuals (either conspecifics or heterospecifics). The utilization of unfamiliar inanimate agents may provide a solution to this problem because this offers the possibility to control subjects’ social experience. This way the amount of familiarization with the potential social partners can be manipulated systematically. Only such well-controlled methods can provide us with information about the cues and triggers of the social evaluation system.

### Cognitive Mechanisms in Social Evaluation

It would be important to determine the decision rules on which the social evaluation system is based and provide the basis of the computations that it makes but there are several problematic issues.

In case of either negativity or positivity bias, only the antisocial or prosocial behavior of the partner is taken into account, thus independently of the weight of the specific behavior, the evaluation of the partner is unidirectional.

However, the situation is different if the social evaluation system has to integrate both negative and positive inputs. At the moment we have no measures that would suggest whether the negative value of any specific antisocial behavior is higher or lower, than the positive value of prosocial behaviors. Until such values are determined experimentally, it is difficult to provide any estimation on how the social evaluation system functions in different species. In addition, we also do not know how kinship and familiarity may modify individual biases.

Based on the current limited data it is difficult to determine both the cues on which social evaluation is based on, as well as the set of decision rules which are applied by the individuals of different species. However, we suggest that there are several features that could become triggers for the social evaluation system. These could include, for example, the present behavioral tendencies of the other individual and outcome of its actions (positive/negative); the familiarity of the partner; and memories about past interactions.

### The Evolution of Social Evaluation

The evolution of cooperation is of great interest among researchers (Axelrod and Hamilton, 1981; Dugatkin, 1997; Sachs et al., 2004; Nowak, 2006). Considering that evolution is based on the survival of the fittest and individuals compete with each other, it is somewhat surprising that cooperation is widespread in the animal kingdom. Nowak (2006) suggested five mechanisms for the evolution of cooperation: kin selection, direct and indirect reciprocity, network reciprocity and group selection. Despite differences among these mechanisms they all can be described by the cost-benefit ratio of cooperation and defection. Cooperative social species may also different in relying on individual recognition on which social evaluation is based. So far there is no evidence that individual recognition plays a specific role in cooperative interactions in social insects, while experimental support for social evaluation in many vertebrates (see above) involves that group mates recognize each other as individuals. Thus in the latter case individuals need to observe and remember the previous interactions of others to be able to evaluate them and adjust their own behavior toward others. In contrast, cooperation in social insects is mostly based on recognition of kinship which makes it less likely that social evaluation evolves.

### The Development of Social Evaluation

We argue that negativity and positivity bias emerges independently during development and eventually is integrated into a cognitive capacity, termed social evaluation. The earlier an individual avoids harmful partners the better, however, showing preference toward prosocial individuals is not necessarily crucial.

Although our main concern is about using third-party information to evaluate social partners, in terms of development (gaining experience) the role of direct information is probably more significant. Direct interactions probably have greater influence on individuals because the outcome is more unequivocal. These experiences may actually help to process information provided by observing third-party interactions (social eavesdropping). During direct interactions individuals can associate the observed behaviors of the other party with the negative or positive outcome on themselves and later recognize these behaviors in a third-party interaction as well, and predict the future behavior of a potential partner without the potential costs of direct experiences. A somewhat similar cognitive scenario was envisaged for human infants to explain how they come to recognize the similarity between themselves and others (“like me” theory developed for human infants; Meltzoff, 2005, 2007a,b). For the transition from direct to third-party evaluation a cognitive system would need to recognize (1) the similarity between the individual’s own actions and the actions of the other (action representation); (2) the similarity between its own reaction to the other’s action and the reaction of others to similar actions, and perhaps (3) being able to evaluate these effects in terms of having negative and positive effect on some mental states.

Learning about antisocial and prosocial behavioral tendencies of partners may be facilitated by specific genetic predispositions, the role of which may be larger in the case of the negativity bias because of the greater cost involved. For example, Sackett (1966) found that rhesus macaques (*Macaca mulatta*) at the beginning of 2 months of age show high frequency of disturbance (rocking, huddling, self-clasping, fear, and withdrawal) to the picture of a threatening monkey. Furthermore, when they were able to control which picture would be projected, threat pictures were turned on less frequently. He concluded that rhesus macaques are predisposed to display fear at the sensitive period in development even in the absence of previous experience.

### Analogies on Evolutionary and Ontogenetic Scales

The emergence of social evaluation can be presented on a timeline that might be analogous on evolutionary and ontogenetic timescales (**Figure 1**). Although in non-human animals there is a lack of data on the emergence of social evaluation, at least results in humans show that the emergence of negativity bias (at 3 months) is followed by the manifestation of positivity bias (at around 6 months) (Hamlin et al., 2010; Hamlin, 2014a). Although the behavioral significance of these early skills is less clear (infants at that age do not have the physical possibility to make a choice between others), a full-fledged social evaluation system may be at work by 5–6 months of age in humans.

Based on the ecological significance (larger effect on fitness) and a single study in capuchin monkeys (Anderson et al., 2013a), which used a comparable method to that applied in human infants (Hamlin and Wynn, 2011), we predict that negativity bias may be more widespread among non-human animals, and evolved also earlier. Testing different species and manipulating their social experience could provide some insight about the relative contribution of genetic and developmental factors to social evaluation.

### Modeling the Mechanism of Social Evaluation

Most experimental studies have been aimed at showing the presence or absence of social evaluation at a certain age (in humans) or in non-human animal species. Little attention has been devoted to mechanisms. Further methodological problem is that in infants spontaneous testing of social evaluation was preferred, while in non-human animals more or less training was applied before testing for social evaluation.

We have suggested that individuals may assign different partnership values to others based on obtaining evidence by either direct or indirect interactions. At some point later in ontogeny they may form specific categories for ‘preferred/prosocial’ and ‘non-preferred/antisocial’ individuals with whom they have extensive experience. Unfortunately, most experimental setups test only for short term social evaluation in which the subject is exposed to a single scenario with unfamiliar partners. However, there is some experimental evidence that infants are also able to calculate relative partnership values when they prefer the antisocially behaving (basically prosocial) partner if it punishes an antisocial partner (Hamlin et al., 2011). Some authors further suggested that individuals might be able to weight the actions of the partners based on their importance and/or reliability as well. Thus some behavior, depending on the situation it was performed in, can count as a better predictor of the individual’s traits than others. For example, Skowronski and Carlston (1987) found that adult humans rely more on negative behaviors in case of morality related context and that extremely negative behavior receives more weight than moderate negative behavior.

**FIGURE 2:**
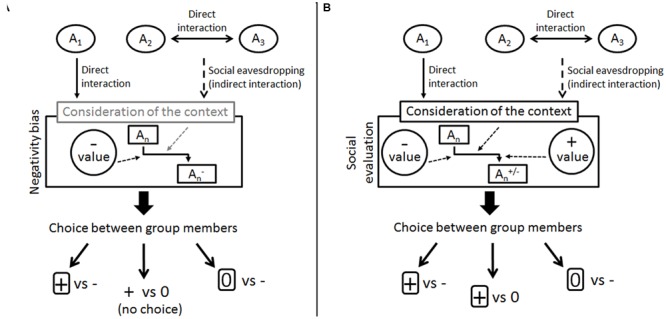
**Two models of negativity and positivity bias and the emergence of social evaluation. (A)** This model is based on Anderson et al.’s (2013a) results in capuchin monkeys; contextual evaluation is only hypothetical, there is no evidence in the study. **(B)** Simple model of social evaluation based on studies by Hamlin et al. (2007) in human infants from around 6 months of age. *A*_n_ stands for the members of the individual’s group. The social evaluation system of the individual cumulates the negative and positive values that are assigned to specific behavior of *A*_n_ in a given context. The individual chooses between the group members based on their overall partnership value.

Based on this, we have developed a simple descriptive model for social evaluation which may provide a common ground for comparative experiments (**Figure 2**). In this model, individuals assign negative or positive values to the behaviors performed by other individuals in direct or indirect interactions. Novel information about their behavior shapes the mental representation of them. Social evaluation manifests in the individual’s choice of the neutral or the prosocial partner depending on the available partners and whether both positivity and negativity bias can be detected or only one of them is present (for example, if only negativity bias is present, the individual do not choose between a neutral and a prosocial partner). Considering that the outcome of an interaction itself is not necessarily the best predictor of the future behavior of others some species may also be able to take into account the context as well (e.g., negative behavior can have positive value based on the context it was performed, see Hamlin et al., 2011).

The results of formerly described studies show that similar phenomena can be detected in different taxa and common evolutionary origin of these cannot be excluded entirely, however, methodological differences disallow any stronger conclusion. Anderson et al. (2013a) suggested that based on their results the capacity to socially evaluate others based on information obtained in third-party interaction might have been present in the common ancestor of Old World and New World monkeys as well (based on our criterion it only applies to negativity bias). However, they did not exclude the possibility that similar phenomena in human infants and capuchin monkeys are the result of convergent evolution. As we suggested earlier, due to its significant effect on survival showing avoidance to antisocial partners may be more widespread, while positivity bias may emerge only in specific social environments. Considering our initial hypothesis that negativity and positivity bias develops independently it may have different origins from an evolutionary point of view.

## Conclusion

Here, we defined social evaluation as the integrated behavior and cognitive system of negativity and positivity bias, and proposed that evaluation based on negative or positive aspects of behavior may have different evolutionary and ontogenetic origins. We further suggest that discrimination between prosocial and antisocial partners in cooperative context is likely to be widespread among species living in long-term closed social groups. Results of studies conducted with non-human mammals and observing interactions among client and cleaner reef fish suggest that negativity and/or positivity bias can be detected in species other than humans. However, we have no clear evidence whether non-human animals are able to show such preference only upon spontaneous observation of third-party interaction.

By conducting studies with a wider range of species having different phylogenetic and ecological backgrounds, we could obtain important information about the origin and development of social evaluation. Considering that in humans social evaluation is discussed in relation to morality, it would be important to find out more details about the relative contribution of genetic and environmental factors to social evaluation.

## Author Contributions

JA, ÁM made substantial contribution to the conception of the work, drafted the work and revised it critically and approved it for publication.

## Conflict of Interest Statement

The authors declare that the research was conducted in the absence of any commercial or financial relationships that could be construed as a potential conflict of interest.

## Footnotes

1 Although the results of the first test trial (in which dogs only had indirect information about the partners) was not reported in the original study, by using the data reported in the *Appendix* of Chijiiwa et al. (2015) (**Table 1**) we found that there was no significant difference in any of the conditions (Binomial GLMM; condition × trial: *p* = 0.870; *trial 1* in case of Helper condition vs. Non-helper condition: *p* = 0.5; Helper condition vs. Control condition: *p* = 0.5; Non-helper condition vs. Control condition: *p* = 1.0).
